# The Association of PON1 192 Q/R Polymorphism with the Risk of Idiopathic Male Infertility in Northern Iran

**Published:** 2019

**Authors:** Setareh Behrouzi, Farhad Mashayekhi, Mohammad Hadi Bahadori

The Editorial Board of *Avicenna Journal of Medical Biotechnology (AJMB)* has decided to retract the original article entitled “The Association of PON1 192 Q/R Polymorphism with the Risk of Idiopathic Male Infertility in Northern Iran” published in the October-December 2018 issue due to a fact which is contrary to the scientific rules and regulation of AJMB.

